# The consequences of abuse, neglect and cyber-bullying on the wellbeing of the young

**DOI:** 10.1371/journal.pone.0327456

**Published:** 2025-08-19

**Authors:** David G. Blanchflower, Alex Bryson

**Affiliations:** 1 Department of Economics, Dartmouth College, Adam Smith Business School, University of Glasgow, IZA, GLO and NBER; 2 Professor of Quantitative Social Science Social Research Institute, University College London; Universiti Putra Malaysia, MALAYSIA

## Abstract

We map changes in the mental health of young people in the United States. Then we run multivariate analyses to capture the independent correlation between young people’s mental health and difficulties experienced in childhood - Adverse Child Experiences (ACE)s – together with the role played by social media, including via cyber-bullying. In doing so we exploit repeat cross-sectional data from the Behavioral Risk Factor Surveillance System (BRFSS) surveys from 2009–2023, the National Health Interview Surveys 1997–2021, the Healthy Minds Surveys 2007–2023 and the Youth Risk Behavior Surveillance System (YRBSS) for high school students. We find that poor mental health is on the rise in the United States, particularly among young women. ACEs have also been on the rise for the young and are strongly and significantly associated with poor mental health in adulthood. The impact of living with a household member with poor mental health is large relative to other ACEs and is particularly pronounced among younger people. Being bullied, including electronically, is also strongly negatively associated with the wellbeing of high school students. In the YRBS we find that time spent in front of a screen has been rising over time for high school children, especially girls, and that this has an independent negative impact on their mental health over and above bullying and being sexually abused as a child.

## 1. Introduction

Recent research indicates that mental health has been deteriorating in the United States since shortly after the Great Recession, particularly among the young [1][2] as well as in Australia [3], Canada [4], Norway [5], the Netherlands [6], and Iceland [7]. It continued to deteriorate during COVID, which was also a huge negative economic shock, but it seems to have started pre-Covid.

It seems that both the U-shape in happiness, found in 145 countries [8], and the hump shape in unhappiness [9] have now gone [10]. Since 2020 data from Global Minds indicates wellbeing has improved with age and ill-being has declined in age in 36 countries across the world [1].

One possible reason for the deterioration is the state of the labor market. Graduating from college in a recession negatively impacts lifetime earnings [11]. The young ages 16–24, according to the Bureau of Labor Statistics were especially impacted by unemployment which peaked in the United States at 18.4% in July 2010 and didn’t drop into single digits until February 2017. This may also have spilled over into a ‘scarring’ effect on their mental health. However, as we shall show in this paper, the decline in mental health appears to have affected those who have yet to enter the labor market too, potentially explaining the poor PISA performance of high school students in the United States. PISA math test scores in 2022 were among the lowest ever measured. Compared to 2012 the proportion of age 15 high school students scoring below a baseline PISA proficiency increased by eight percentage points. ^(^PISA 2022 Results – USA, OECD, 5^th^ December 2023. https://www.oecd.org/publication/pisa-2022-results/country-notes/united-states-a78ba65a/)

The depth of the mental health crisis is apparent in suicide rates among those aged 15–24 which rose sharply in the US from around 2007 and especially so for young women [12][13]. From 2011 to 2021 suicide rates of women ages 15–24 doubled while those of young men rose by a half, although they are still around four times higher than for young females who attempt more but succeed less. Suicide rates/100000 for ages 15–24 were as follows for men with women in parentheses 2007 = 15.7 (3.1); 2011 = 17.6 (4.0); 2015 = 19.4 (5.3); 2017 = 22.7 (5.8); 2019 = 22.0 (5.5); 2020 = 22.4 (5.8); 2021 = 23.8 (6.1). https://www.cdc.gov/nchs/data/databriefs/db464-tables.pdf#2.

Using three data sets Udupa et al. (2022) [2] confirm this decline in the mental health of young people, particularly among women. 1) The Behavioral Risk Factor Surveillance Survey (BRFSS), 1993–2020 (n = 8.8 million) 2) the National Health Interview Survey, 1997–2018 (n = 657,000) and3) the National Health and the much smaller Nutrition Examination Survey (NHIS), 2005–2020 (n = 39,467). Speculating as to the reasons they argue COVID is not a primary cause since the trend pre-dates it by many years. They also discount a change in willingness to admit to mental health issues, given that suicide also increased among these age groups over the same period. We extend their work using the Behavioral Risk Factor Surveillance System Surveys (BRFSS), through to 2024, and the Youth Risk Behavior Surveillance System (YRBSS) data from 1999–2023 for high school students ages 14–18. In both, we show a deterioration in mental health especially of women. We also report evidence from the National Health Interview Surveys (NHIS), 1997–2023.

In doing so we build on our earlier work tracking the mental health of the young in the United States. First, in [1] we found that there has been a dramatic decline in the wellbeing of young people under the age of twenty-five, especially young women, in both the US and the UK, but also elsewhere. This decline in mental health occurred from around 2011.

Second, in [14] we used data for Europe and the United States to examine the impact of a number of ACE variables on wellbeing in later life. The datafiles used were the General Social Survey, 1973–2022 and the BRFSS, 2009–2023 for the United States, the Eurobarometer, 2001 and the European Social Survey, 2014. Death of a parent, parental separation or divorce, financial difficulties, the prolonged absence of a parent, quarreling between parents, parental unemployment, sexual assault, experiencing long-term health problems, being bullied at school and being beaten or punched as a child all have long-term impacts on wellbeing. The evidence of ACEs impacting adult wellbeing outcomes was consistent across 50 different wellbeing measures including sixteen positive affect measures (including happiness, life satisfaction and satisfaction with family and social life) happiness; life satisfaction; financial situation; life you lead; family life; social life; leisure life; income; standard of living; health status; time to do things you have to do; consideration shown by others; job satisfaction; pay satisfaction; job security satisfaction and satisfaction with democracy. Plus twenty-six negative affect measures such as the GHQ6, high blood pressure, loneliness, being down and depressed and tired, i.e., lost sleep; feeling unhappy; could not overcome difficulties; strain; worthless; high blood pressure; not valued; no friends; left out of my family; left out of society; not useful; some people look down on me; fear poverty; confidence; stress; pain; little pleasure in doing things; down, depressed or hopeless; felt a failure; trouble concentrating; bad mental health days; bad physical health days; depressive disorder; anxiety disorder; distress; tired or little energy. In addition, we find childhood adversity impacts views on the area where the respondent lives in eight variables, including unemployment, drugs, violence and vandalism plus democracy in their country. In relation to the local neighborhood – noise; unemployment; violence; drug abuse; vandalism; bad buildings; area you live in and a bad reputation.

Third, in [15] we made use of birth cohort data from the British National Child Development Study of everyone born in Great Britain in one week in March 1958. We use characteristics from childhood including reports from a parent on whether the child was bullied at 7 and/or at age 11 and wellbeing data collected prospectively throughout cohort members’ lives. Perhaps unsurprisingly, bullying makes adolescents at age sixteen more worried, miserable and tearful. But it has persistent effects on wellbeing across the life-course. We show bullying negatively impacts life satisfaction at ages 42, 46, 50 and 62 as well as several other wellbeing measures at ages 42, 50 and 55. It also significantly lowers the probability of having a job as an adult right through to age 62. These effects are independent of other childhood experiences, such as whether the child reported that they got on well with their mother or father when they were 16, many of which also have persistent effects on outcomes in adulthood.

In this paper we document the rise in mental ill-health among the young in the USA since 2009 using BRFSS data plus data from the NHIS, Healthy Minds and from high school students in the YRBSS. The four data sets used in this paper all show the worsening of the mental health of the young. Second, we show that the worsening of youth mental health is associated with an increase in time spent online, especially for those with the worst mental health. Finally, we show that poor mental health is also associated with Adverse Child Experiences (ACEs) which appear to have risen over time, especially for the young. We document a rise in reports among young people that a parent suffered from depression while they were a child. This is particularly the case for young females.

Some studies suggest the rise in mental ill-health for the young coincided with the growth in smart phone usage. The iPhone was unveiled in January 2007 and sold 4.7 million phones in Q32008, and the iPad was launched in January 2010 (https://www.theguardian.com/technology/2012/jan/24/smartphones-timeline).

In q12011 Apple sold 18.6 million iPhones, while Samsung sold 17.5m. Sales of smartphones worldwide rose from 122m in 2007; 297m in 2010; 472m in 2011; 690m in 2012; 970m in 2013; 1.2 billion in 2014 and 1.5 billion a year since 2018 (https://www.sellcell.com/how-many-mobile-phones-are-sold-each-year/)

Twenge and Farley (2020) [16] examined a sample of those ages 13–15 using data from 2015 wave of the UK Millennium Study, a cohort born in 2000/2001 and showed that hours spent on social media and internet use were more strongly associated with mental ill-health than hours spent on electronic gaming and TV watching with girls showing stronger correlations than boys. Twenge and Martin (2022) [17] found from an analysis of YRBSS, and the UK Millennium Study that digital media time was more strongly associated with low well-being among girls than among boys, particularly for smartphone and social media use. We extend that work and show that a rise in screen time has impacted well-being especially among high school girls.

In the YRBSS 1999–2023 we find a big rise in sadness and hopelessness for female high school students matching the rise in despair noted above for those age 18–24. We also present evidence of a rise in screen time usage of young women measured as hours spent in front of a TV, computer, smart phone, or other electronic device watching shows or videos, playing games, accessing the internet, or using social media excluding schoolwork. It does appear to be a significant contributor to the rise in sadness and hopelessness.

## 2. Adverse Childhood Experiences (ACEs)

There is a substantial literature showing that experiences in childhood can impact physical as well as emotional outcomes contemporaneously, but also in adulthood.

Petrucelli, Davis and Berman (2019) [18] in a systematic review of the impact of ACEs found that they had a significant effect on both physical and mental outcomes. Impacts have been shown on diabetes [19], on sleep disorders [20] and on asthma [21]. Hardcastle et al (2018) [22] found a relationship between adverse childhood experiences and educational and employment success in England and Wales. Sacker, Murray, Maughan and Lacey (2023) [23] show a relationship in the UK between poor social care in childhood and adult outcomes which is especially marked for minority children. Some studies have examined the impact of ACEs in other countries including Iceland [24], Eritrea [25] and China [26].

Other childhood trauma also appears to have long lasting effects. Akbulut-Yuksel, Tekin and Turan (2022) [27] examined the impact of early life exposure to warfare on long–term mental health, using data on the quantity of bombs dropped in German cities by Allied Air Forces during World War Two (WWII) using the German Socioeconomic Panel. They found that cohorts younger than age five at the onset of WWII or those born during the war were in significantly worse mental health later in life when they were between ages late 50s and 70s. They conclude that “*children bear the invisible wounds of wars*” (2022, p.23). Other papers show the negative impact of war on adult wellbeing including [28] on the Vietnam War and Kim (2017) [29] on the Korean War who concluded that “*war exposure in late childhood to early teenage years has a long-run negative impact on variables related to mental health, including depression, fear, insomnia, and loneliness*” (2017, p.431). Economic factors matter also. For example, a rise in cocoa prices in early life in Ghana decreased the likelihood of severe mental distress in adulthood [30].

Prenatal exposure to the death of a maternal relative increases take-up of ADHD medications during childhood and anti-anxiety and depression medications in adulthood [31]. In the UK children with higher ACE scores had nearly double the risk of premature mortality through middle age fifty than children without ACE [32]. Brown et al (2009) [33] found that “ACEs are associated with an increased *risk of premature death*”. A Danish study also found ACEs raised mortality [34]. Yu et al (2022) [35] used a US birth cohort to examine 13 individual ACEs assessed between birth and age seven. At the start of the study in 1979 respondents were ages 12–20. They were followed until 2016 – more than three decades later – and observed that 3,344 had died from an overall sample of 46,129. Participants were offspring (born in 1959–1966) of participants enrolled in the Collaborative Perinatal Project. (https://www.archives.gov/research/electronic-records/nih.html) The authors found that “*higher ACE scores led to an increasing risk of premature mortality, with each additional adversity associated with about a 10% higher risk of premature mortality, and exposure to ≥4 ACEs associated with a 45% higher risk of premature mortality*” (p.7).

Some earlier studies have examined the ACE data in the BRFSS that we examine below [36–38]. Li and Penn (2023) [39] used the 2019 and 2020 BRFSS and showed that ACEs were higher for females and did not find a significant difference in ACE scores between the White population and the Black population. They found that ACEs lowered incomes and the probability of being in work in adulthood. Monnat and Chandler (2015) [40] examined the impact on adult health from ACEs using data on 52,250 adults ages 18–64 using pooled 2009–2012 BRFSS data. They found that “*experiencing childhood physical, verbal, or sexual abuse, witnessing parental domestic violence, experiencing parental divorce, and living with someone who was depressed, abused drugs or alcohol, or who had been incarcerated were associated with one or more of the following health outcomes: self-rated health, functional limitations, diabetes, and heart attack*”.

Harter and Harter (2022) [41] used the 2012 BRFSS to examine the impact of ACEs on adult financial wellbeing. They analyzed how respondents’ self-reported levels of food security and housing security are influenced by demographics and ACEs and found that, at various income levels, financial stress in adulthood is related to childhood trauma.

Having introduced the data sets in Section Three, Section Four presents evidence on the declining well-being of the young in the United States since the mid-1990s before moving on to examine the extent to which this is correlated with Adverse Child Experiences. We show that both poor mental health and ACEs have risen over time and that ACEs are strongly correlated with poor mental health in adulthood.

## 3. Data

### 3.1. National Health Interview Surveys 1997-2023 (NHIS)

The National Health Interview Surveys (**https://www.cdc.gov/nchs/nhis/index.htm**) is a national survey conducted by the U.S. Census Bureau on behalf of the National Center for Health Statistics (NCHS). The National Health Interview Survey (NHIS) is the principal source of information on the health of the civilian noninstitutionalized population of the United States and is one of the major data collection programs of the National Center for Health Statistics (NCHS), which is part of the U.S. Centers for Disease Control and Prevention (CDC). We downloaded these data from the IPUMS data service(https://nhis.ipums.org/nhis/).

### 3.2. Healthy minds, 2007–2023 (https://healthymindsnetwork.org)

The Healthy Minds surveys are an annual web-based survey in the United States examining mental health and related factors among undergraduate and graduate students. Institutions choose to participate in the surveys. Publicly available data can be downloaded on application since 2007. A comprehensive list of publications using these data is available here https://healthymindsnetwork.org/publications/

### 3.3. Behavioral Risk Factor Surveillance System (BRFSS)

The BRFSS is the nation’s premier system of health-related telephone surveys collective data about U.S. residents regarding their health-related risk behaviors, chronic health conditions, and use of preventive services. Established in 1984 with 15 states, BRFSS now collects data in all 50 states as well as the District of Columbia and three U.S. territories. BRFSS completes more than 400,000 adult interviews each year, making it the largest continuously conducted health survey system in the world. Annual data files are publicly available here https://www.cdc.gov/brfss/annual_data/annual_data.htm

### 3.4. Youth Risk Behavior Surveillance System, 1999–2023

(https://www.cdc.gov/healthyyouth/data/yrbs/index.htm)

We also have data on the mental wellbeing of high school students ages 14–18 in 9^th^ through 12^th^ grades, in the Youth Risk Behavior Surveillance System (YRBSS) also conducted by the CDC. https://www.cdc.gov/healthyyouth/data/yrbs/index.htm. The data are publicly available for download. The survey, which reports data every odd year, contains data on mental well-being. The CDC has produced a number of reports using the YRBSS [42–49]. It first asked a question on sadness and hopelessness in 1999 that is not dissimilar to the distress and despair variable used earlier from the BRFSS.

## 4. Evidence on the declining mental health of the young, internet use and ACEs

We have data available from each of the four US datasets that consistently show declining wellbeing of the young in the years since the Great Recession. Blanchflower (2025) [10] also documented evidence of declining mental health of the young in 11 other data files – National Health and Nutrition Examination Study, The National Survey of Drug Use and Health, American National Election Study, General Social Surveys, Gallup World Poll, the Global Flourishing Survey, Global Minds, US Household Pulse Surveys, the National Survey of Children’s Health, OECD PISA Surveys and the All-payer IQVIA Longitudinal Prescription Database.

We start by outlining trends in youth mental health and then examine the role of ACEs before moving on to look at the impact of screen time and the internet before turning to broader implications in the final section.

### 4.1. Trends in youth mental health

#### 4.1.1. National Health Interview Surveys 1997-2023 (NHIS).

We examine the NHIS through to 2023, extending work previously undertaken by [2] who showed declining mental health from 1997–2018. Udupa et al (2020) [2] used the 6-item Kessler scale which is based on the six items below where participants were asked:

*Q1. “During the past 30 days, how often did you feel … _1) so sad that nothing could cheer you up, 2) nervous, 3) restless or fidgety, 4) hopeless, 5) that everything was an effort, 6) worthless. Response choices were recoded as: “all of the time” = 4, “most of the time” = 3, “some of the time” _ = 2, “little of the time” = 1, and “none of the time” = 0.*

The six items are added together to get the Kessler score which ranges between 0 and 24 where a higher score denotes poorer mental health. Mean scores on each item are all less than one with the majority of respondents answering, ‘none of the time”. Weighted means with percent saying none of the time in parentheses were as follows – Sadness = .39 (76%); Hopeless = .22 (88%); Everything an effort = .48 (75%); Nervous = .59 (64%); Worthless = .17 (90%) and Restless = .61 (66). Kessler mean = 2.45 (48%).

**Fig 1:**
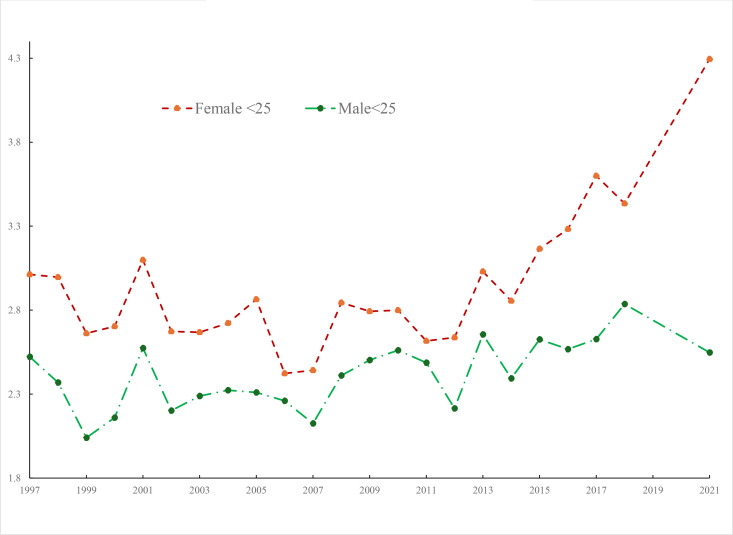
Kessler score NHIS by age.

Fig 1 plots these data over time using sample weights for those age 25 and over along with those under 25 by gender (n = 685,640). We extend the analysis through 2021. The incidence of poor mental health, on this measure, has been trending up a little for those aged 25 and over (not shown) and for males aged under-25 but it has risen dramatically for young women aged under-25 since around 2011.

The 2023 survey does not have the Kessler score available but does have four additional negative affect questions.

*Q2. How often little interest in things, past 2 weeks*

*Q3. How often feeling down, past 2 weeks*

*Q4. How often felt nervous/anxious/on edge, past 2 weeks*

*Q5. How often can’t stop/control worrying the past 2 weeks*

*Not at all (=1), several days (=2), More than half the days (=3), Nearly every day (=4).*

It also contains a life satisfaction question.

*Q6. In general, how satisfied are you with your life? Are you very satisfied (=4), satisfied (=3), dissatisfied (=2), or very dissatisfied (=1)?*

We run simple OLS regressions, controlling for race, region, education and gender to capture the correlation between age category and these measures of wellbeing and ill-being. Table 1 shows the results of estimating these five equations. The first four show quite clearly that ill-being on all four measures declines in age. This is not the case in the final column for life satisfaction which declines in age through to age 65. This is consistent with our earlier work where the poor mental health of the young is more apparent in negative affect measures [10]. In these data, women tend to have poorer mental health than men, but they also express higher life satisfaction.

**Table 1:** Determinants of Ill-being, National Health Interview Survey, 2023. Age < 75.

	Little interest	Feeling down	Felt nervous/anxious	Can’t stop worrying.	Life satisfaction
Female	.0189 (2.19)	.0528 (6.48)	.1341 (13.59)	.1148 (12.64)	.0219 (2.94)
25-34	.0171 (0.89)	.0151 (0.85)	−.0304 (1.40)	.0125 (0.63)	−.0521 (3.15)
35-44	−.0506 (2.66)	−.0338 (1.89)	−.1453 (6.70)	−.0596 (2.99)	−.0418 (2.54)
45-54	−.0536 (2.77)	−.0376 (2.07)	−.2147 (9.75)	−.0775 (3.83)	−.0311 (1.86)
55-64	−.0954 (5.10)	−.0932 (5.30)	−.3004 (14.10)	−.1592 (8.12)	−.0112 (0.69)
65-74	−.2054 (10.76)	−.1888 (10.53)	−.4819 (22.16)	−.2858 (14.29)	.0480 (2.91)
Constant	1.3641	1.3487	1.6616	1.3988	3.2835
Pseudo R^2^	.0297	.0288	.0539	.0372	.0338
N	24,577	24580	24575	24572	25232

Notes: Excluded age < 25; whites. T-statistics in parentheses. Controls include race, Hispanic status, region, education and gender refused and DK.

#### 4.1.2. Healthy Minds, 2007-2023 (HM).

Lipson et al (2022) [50] examined these data through to 2021. Their sample included students aged twenty-five and above which accounted for around a quarter of students. They reported a rise in poor mental health based on a 9-item depression score and a 7-item anxiety score. Each item allowed respondents to report 0 “not at all’; 1 “several days’ 2 = more than half the days and 3 nearly every day. The estimates they reported related to those scoring at least ten on these scales with both showing a sharp rise.

**Table:** 

	Depression	Anxiety
2013	17	17
2014-2015	20	20
2015-2016	26	21
2016-2017	31	26
2017-2018	37	32
2018-2019	37	32
2020-2021	41	35

We have access to two more recent surveys, 2021–2022 and 2022–2023 plus surveys from 2007–2012 and decided to examine changes over time by gender in one of the component variables which asked the following.

*Q7. Over the last two weeks how often have you been bothered by any of the following problems? Feeling down, depressed or hopeless? – not at all (=1), several days (=2), more than half the days (=3), nearly every day (=4).*

We grouped years together for the earlier years due to smaller sample sizes. We also restricted the sample to those under the age of twenty-five. Table 2 reports weighted distributions by gender as well as a mean score using the scoring 1–4 for twelve year-groupings. The main finding is the increase over time especially for young women. For example, 28% of females in 2022–2023 reported feeling down, depressed or hopeless more than half the days or nearly every day versus 14% in 2012–2013. For males 20% said this up from 13% in 2012–2013. It is also notable that females have higher mean depression scores than men. They are also more likely to report higher frequencies of experiencing depression. The mental health of the young and especially young women appears to have deteriorated in the last dozen years or so. We replicate this result in other datasets examined below.

**Table 2:** Over the last two weeks how often have you been bothered by any of the following problems? Feeling down, depressed or hopeless.

	Not at all	Several days	> half	Nearly every	Average score	N
Males %						
2022-2023	38	42	11	9	1.91	14,015
2021-2022	34	42	14	10	2.00	17,219
2020-2021	36	41	14	9	1.96	24,772
2019-2020	37	42	12	9	1.93	20,074
2018-2019	39	39	13	9	1.93	11,414
2017-2018	37	40	13	10	1.95	12,062
2016-2017	43	39	11	7	1.81	9,945
2015-2016	46	40	9	5	1.73	8,182
2014-2015	48	38	9	5	1.71	7,080
2012-2013	48	38	9	4	1.69	14,618
2010-2011	45	42	9	5	1.73	11,326
2007-2009	47	41	8	4	1.68	4,924
Females %						
2022-2023	27	45	17	11	2.12	3,885
2021-2022	25	45	18	13	2.18	44,085
2020-2021	26	44	17	13	2.17	64,009
2019-2020	29	46	16	10	2.06	43,501
2018-2019	30	44	16	10	2.06	23,371
2017-2018	30	45	16	10	2.05	26,349
2016-2017	34	45	14	7	1.94	21,614
2015-2016	38	46	11	6	1.85	15,770
2014-2015	42	42	11	6	1.81	8,713
2012-2013	44	42	10	4	1.75	27,089
2010-2011	40	45	10	4	1.78	20,598
2007-2009	39	47	9	5	1.79	8,961

Notes; 1 = not at all, 2 = several days 3 = more than half the days, 4 = nearly every day. Source: Healthy Minds, 2007–2023

#### 4.1.3. Behavioral Risk Factor Surveillance Surveys, 1993–2023.

We have data in the BRFSS from 1993–2023 on the number of bad mental health days in the last thirty. The exact question asked is:

*Q8. Now thinking about your mental health, which includes stress, depression and problems with emotions, for how many of the past 30 days was your mental health not good?*

**Fig 2:**
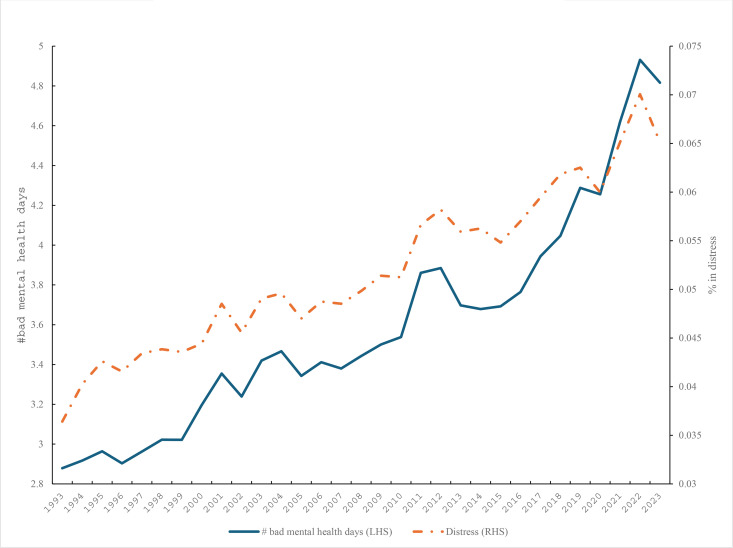
Mental health, 1993-2023, BRFSS.

The question has been asked in the BRFSS since 1993 [44] and can be used to construct a mental distress/despair (1,0) dummy variable where the respondent reports all 30 days are bad mental health days. Blanchflower, Bryson and Xu (2024) [1] examined the same BRFSS data for the young – see also [43]. The number of bad mental health days and the mental distress variable are plotted in Fig 2. Both mental health days and distress rose steadily over time though the rate of decline in mental health appears to have increased in the last decade.

Included in the BRFSS data files is also an equivalent question that relates to bad physical health days. The question is:

*Q9. “Now thinking about your physical health, which includes physical illness and injury, for how many days during the past 30 days was your physical health not good?”*

We use that variable to construct a measure of bad physical distress which is where the respondent reports all thirty days were bad physical health days, zero otherwise.

**Fig 3:**
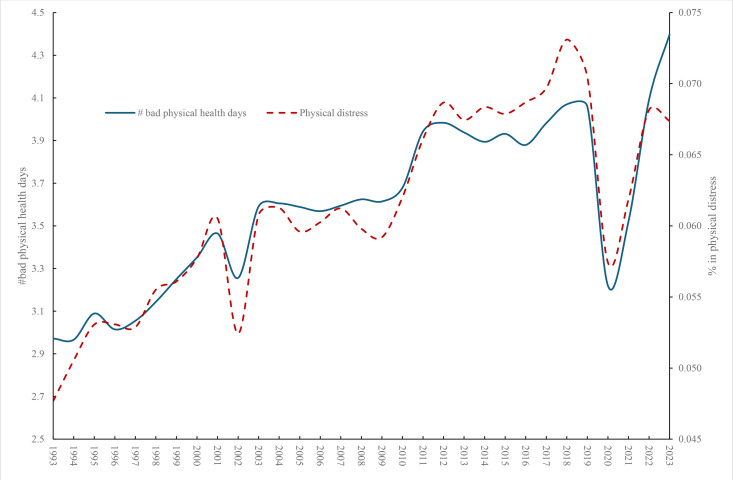
Physical health, 1993-2023, BRFSS.

Fig 3 plots both the average number of physical health days and the incidence of physical distress. The number of bad physical health days and distress rose through 2010 but in contrast to mental health then remained broadly steady before declining in 2020 during COVID and then picking up subsequently. The mean number of bad physical health days in 2022 was 4.09 compared with 4.93 for bad mental health days and.068 for physical distress and.070 for mental distress. In the case of young people there was a much more marked difference between physical and mental health incidence. The mean number of bad physical health days for those under 25 in 2022 was 2.72 compared with 7.58 for bad mental health days and.026 for physical distress and.090 for mental distress.

**Fig 4:**
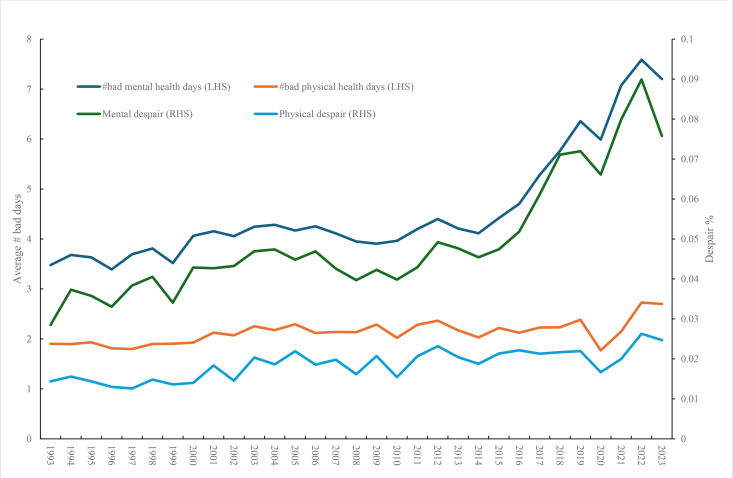
Mental and physical well being age < 25, BRFSS.

Fig 4 restricts the BRFSS trend analysis to under-25s. It includes both physical and mental health days and the two despair variables defined as 30 of 30 days in the month being ‘bad’. Bad physical health days and physical despair are broadly flat, with both showing dips in COVID in 2020. In contrast both bad physical health days and mental despair rise steadily from around 2011 before dropping in 2023.

**Fig 5:**
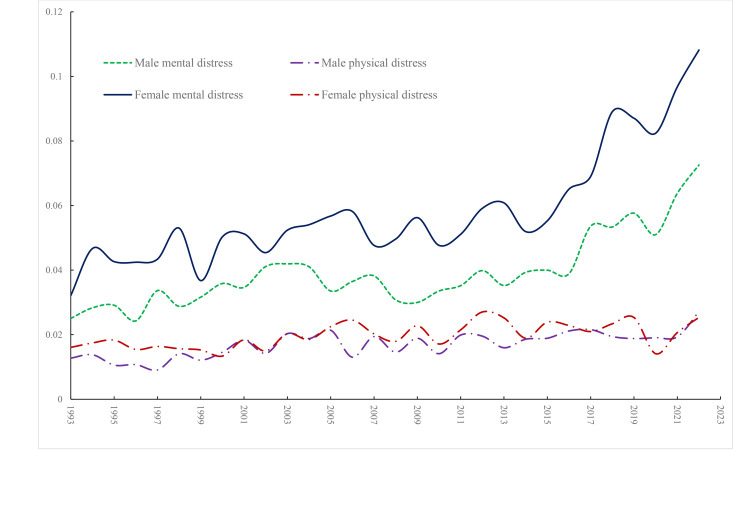
Mental and physical distress % by gender age under 25, BRFSS.

**Fig 6:**
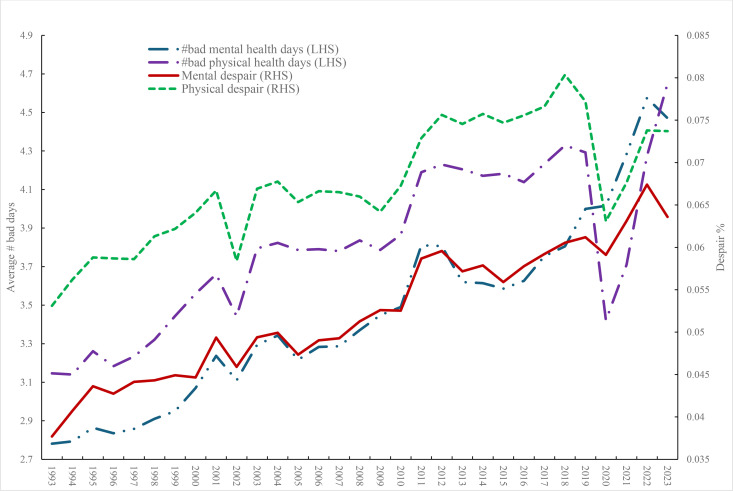
Mental and physical well being age 25 and over, BRFSS.

Fig 5 focuses on physical and mental distress for young men and women separately. Physical distress is flat for males and females, but mental distress rises for both and is consistently higher for women than men. For example, in 2022 10.8% of young women suffered mental distress compared to 7.3% of young men while physical distress affected 2.7% and 2.5% respectively. Whereas mental distress is more evident than physical distress among the young it is the other way round for those aged 25 and over where the physical lines are above the mental lines until the end of the period (Fig 6). Physical despair is always above mental despair. All trend up slightly over time.

### 4.2. The role of adverse child experiences

#### 4.2.2. ACEs in the BRFSS.

We also have data in the BRFSS on eight variables relating to adverse experiences in childhood (ACEs). We now update evidence in [15], with a pooled data file for the nine surveys of 2009–2012 and 2019–2024 from BRFSS using the same module on adversity in childhood. There are an additional 878 observations from 2013 obtained from the 2012 survey and 1027 in 2024 from the 2023 survey. Sample sizes are 2009 = 11,239; 2010 = 22,846; 2011 = 45,486; 2012 = 28,573; 2019 = 94,816; 2020 = 119627, 2021 = 53,308; 2022 = 45,660; 2023 = 50,702. Total with ACE observations = 474,162. Thirty-nine states provided information on Adverse Childhood Experiences – Alabama; Arizona; Arkansas; Delaware; District of Columbia; Florida; Georgia; Hawaii; Idaho; Indiana; Iowa; Kentucky; Louisiana; Michigan; Minnesota; Mississippi; Missouri; Montana; Nevada; New Hampshire; New Jersey; New Mexico; North Carolina; North Dakota; Oklahoma; Oregon; Pennsylvania; Rhode Island; South Carolina; South Dakota; Tennessee; Texas; Utah; Vermont; Virginia; Washington; West Virginia; Wisconsin and Wyoming. Details are provided in the appendix.

Each of the nine surveys contain information on eight adverse childhood experiences that respondents reported as an adult on events that occurred when they were a child. There were three other questions we decided not to use as we found they had little statistical impact once the eight others were included*. a) Did you live with anyone who served time or was sentenced to serve time in prison, jail or other correctional facility? Yes/No = .093 b). How often did your parents or adults in your home ever slap, hit, kick, punch or beat each other up? Never, once or more than once? = .174. C) How often did a parent or adult in your home ever swear at you, insult you or put you down? Never, once or more than once?*.35. The weighted means are based on setting the last two variables to dummy variables with once or more than once set to zero, never = 0. See for example 2010 BRFSS Questionnaire (https://www.cdc.gov/brfss/questionnaires/pdf-ques/2010brfss.pdf)

Four events relate to their parents and four to whether they were abused, including sexually in childhood. The questions used were as follows with overall weighted means across all year in parentheses.

“*I’d like to ask you some questions about events that happened during your childhood. This information will allow us to better understand problems, that may occur early in life and may help others in the future. This is a sensitive topic, and some people may feel uncomfortable with these questions. At the end of this section, I will give you a phone number for an organization that can provide information and referral for these issues. Please keep in mind that you can ask me to skip any question you do not want to answer. All questions refer to the time period before you were 18 years of age. Now, looking back before you were 18 years of age…*

*Q10. Did you live with anyone who was depressed, mentally ill, or suicidal? (18.5%).*

*Q11. Did you live with anyone who was a problem drinker or alcoholic? (24.1%).*

*Q12. Did you live with anyone who used illegal street drugs or who abused prescription medications? (11.8%).*

*Q13. Were your parents separated or divorced? (30.4%)*

*Q14. How often did anyone at least 5 years older than you or an adult, ever touch you sexually? (11.5%)*

*Q15. How often did anyone at least 5 years older than you or an adult, try to make you touch them sexually? (8.6%).*

*Q16. How often did anyone at least 5 years older than you or an adult, force you to have sex? (5.2%).*

*Q17. Before age 18, how often did a parent or adult in your home ever hit, beat, kick, or physically hurt you in any way? Do not include spanking. (23.3%).*

The three variables relating to sexual abuse (Q5-Q7) allowed the possibility of responding never, once or more than once. For simplicity we recoded all of these variables as (1,0) Yes/No dummies. We used the tetrachoric command in stata for binary variables.The correlation matrix is as follows (n = 471,081).

**Table:** 

	Depress	Drink	Drugs	Divorce	Touch	Them	Hurt
Depression	1.0000						
Drink	.2866						
Drugs	.3320	.3235					
Divorce	.2019	.2438	.2333				
Touch	.2322	.1955	.1913	.1530			
Them	.2146	.1783	.1919	.1486	.6900		
Hurt	.2526	.2431	.2076	.1825	.2314	.2170	
Have sex	.1860	.1616	.1769	.1322	.5356	.5622	.2041

All eight ACEs are positively correlated with each other with coefficients ranging from.18 to.56. We undertook exploratory factor analysis to establish whether the items loaded on one or more underlying factors. A single factor emerged with an eigenvalue above one (2.2) indicating that all eight ACEs are associated with a single underlying construct. The Cronbach alpha of 0.70 is an acceptable reliability score. We then proceed initially to sum the ACEs together and create a variable scored from zero to eight (n = 471,081) with the weighted distribution as follows.

**Table:** 

	Male	Female	Male <25	Female <25
0	49.3	44.2	42.5	34.1
1	24.4	22.7	26.1	23.7
2	11.6	11.8	13.3	12.6
3	6.8	7.9	7.9	10.0
4	4.0	5.3	4.9	7.7
5	2.4	3.7	3.5	5.9
6	0.8	2.1	1.1	2.7
7	0.5	1.4	0.5	2.1
8	0.3	0.8	0.3	1.2
Mean	1.07	1.39	1.25	1.80

Around four in ten of both men and women and a third of young women say they had no ACEs. One in five young women have four or more ACEs versus one in ten of young men, leading to a much higher mean among young women (1.80 versus 1.25). Also using BRFSS, Swedo et al (2023) [36] identified 11 ACEs and found 64% of respondents reported one or more of these eleven ACEs. Looking at our smaller subset of eight ACEs, we find that 51% of males and 56% of females had one or more ACEs.

Mean weighted rankings of the average number of ACEs by state was as follows in order: Oregon = 1.70; Nevada = 1.58; New Mexico = 1.48; Missouri = 1.41; Michigan = 1.40; Utah = 1.40; Kentucky = 1.39; New Hampshire = 1.39; Indiana = 1.38; Montana = 1.38; Washington = 1.37; South Carolina = 1.36; Wyoming = 1.36; Arkansas = 1.35; Tennessee = 1.35; Arizona = 1.35; Idaho = 1.33; Florida = 1.32; Alabama = 1.32; Delaware = 1.29; Pennsylvania = 1.28; Rhode Island = 1.26; West Virginia = 1.26; Georgia = 1.24; Virginia = 1.23; Mississippi = 1.22; Texas = 1.21; Iowa = 1.19; Oklahoma = 1.18; North Dakota = 1.18; DC = 1.16; Wisconsin = 1.14; Vermont = 1.10; South Dakota = 1.10; North Carolina = 1.09; Hawaii = 1.06; Louisiana = 1.04 and Minnesota = .98.

Parts a and b of Table 3 show the incidence of the eight different ACEs by gender and race. The most common is parental divorce which is high for the young. As we show in part c of Table 3, there is evidence of a rise over time in the incidence of ACEs, the reasons for which are unclear. The biggest rise is for parents being depressed, especially among the young – up from 23% in 2009–2013 to 31% in 2019–2024. Parts d and e show that there has been a rise in the incidence of ACEs for the young between the first time period (2009–2013) and the second (2019–2024) with this being especially apparent for young females. Of particular note is the rise in the proportion who report that a parent was depressed – up from 28% in the first period to 39% in the second versus 21% and 29% for young men. There was also a substantial increase in reports of young women being asked to touch others sexually, up from 8% to 13%. The number of Aces also rose sharply for young women up from 1.57 to 1.89.

**Table 3:** ACES in the BRFSS, 2009-2013 and 2019-2024.

a)	All	Male	Female	White	Non-white
Depressed	18	15	21	20	15
Drinker	23	21	25	24	21
Drugs	11	11	11	11	11
Divorced?	30	30	30	27	36
Hit	17	16	18	16	20
You touched	11	6	16	11	11
Touch them	8	5	11	8	9
Sex	5	3	7	4	6
Aggregate	1.32	1.07	1.39	1.21	1.23
N	474,203	209,206	264,997	378,401	95,931
b)	Age < 25	Age≥25	Males <25	Females <25	
Depressed	29	17	24	35	
Drinker	23	23	21	27	
Drugs	17	11	16	19	
Divorced?	41	29	39	44	
Hit	18	17	15	21	
You touched	10	11	5	16	
Touch them	8	8	4	12	
Sex	5	5	2	8	
Aggregate	1.51	1.20	1.25	1.82	
N	21,848	452,355	11,737	10,111	
*c)*	*2009-2013*	*2019-2024*	*2009-2013*	*2019-2024*	
	*Age≥25*	*Age≥25*	*Age < 25*	*Age < 25*	
Depressed	16	19	23	31	
Drinker	24	23	23	24	
Drugs	10	11	17	17	
Divorced?	26	31	38	42	
Hit	16	17	16	18	
You touched	9	11	7	11	
Touch them	7	9	5	8	
Sex	4	5	4	5	
Aggregate	1.12	1.25	1.32	1.57	
N	109,063	365,140	4,406	17,442	
d)	Age < 25 male		Age < 25 female		
	2009−13	2019-2024	2009−13	2019-2024	
Depressed	21	29	28	39	
Drinker	21	22	25	28	
Drugs	16	16	19	19	
Divorced?	37	39	41	44	
Hit	12	15	19	22	
You touched	2	5	11	17	
Touch them	2	4	8	13	
Sex	1	2	6	8	
Aggregate	1.12	1.33	1.57	1.89	
N	2249	11068	2249	8954	
e)	Age≥25 male		Age ≥25 female		
	2009−13	2019-2024	2009−13	2019-2024	
Depressed	12	13	17	18	
Drinker	21	20	25	24	
Drugs	7	9	7	9	
Divorced?	19	24	20	24	
Hit	14	14	15	16	
You touched	5	6	13	15	
Touch them	4	5	9	10	
Sex	2	2	5	6	
Aggregate	0.85	0.92	1.11	1.21	
N	41663	154,350	62994	190,768	

*Q1. Did you live with anyone who was depressed, mentally ill, or suicidal?*

*Q2. Did you live with anyone who was a problem drinker or alcoholic?*

*Q3. Did you live with anyone who used illegal street drugs or who abused prescription medications?*

*Q4. Were your parents separated or divorced?*

*Q5. How often did anyone at least 5 years older than you or an adult, ever touch you sexually?*

*Q6. How often did anyone at least 5 years older than you or an adult, try to make you touch them sexually?*

*Q7. How often did anyone at least 5 years older than you or an adult, force you to have sex?*

*Q8. Before age 18, how often did a parent or adult in your home ever hit, beat, kick, or physically hurt you in any way? Do not include spanking. (23.3%).*

Table 4 reports six OLS regressions modelling the determinants of the number of ACEs. As we move to the right, controls are added. The first column includes age, gender and race and year. The second adds state, the third adds education and labor force status and the final two columns relate to the young. The young have the highest incidence; the number of ACEs declines in age. There is a good deal of stability in the coefficients as controls are varied. Females have a higher number of ACEs than men. ACEs are higher for native Americans confirming earlier research [51]. The final two columns report estimates for those under age twenty-five, and has especially large female and time effects which we explore further below. For both the young, and overall, a female*time variable has a significantly positive coefficient so the rise in the number of ACES over time is higher for women.

**Table 4:** Determinants of adverse childhood experiences aggregates OLS, 2009-2013 and 2019-2024.

Female	.3311 (75.10)	.3300 (75.09)	.3342 (75.23)	.2916 (27.53)	.6093 (26.81)	.0173 (3.00)			
Time	.0151 (29.43)	.0165 (19.20)	.0189 (22.31)	.0164 (15.78)	.0208 (4.43)	.0125 (2.28)			
Female *time				.0045 (4.43)		.0173 (3.00)			
25-34	.0594 (4.94)	.0529 (4.42)	.0653 (5.16)	.0653 (5.16)					
35-44	−.0769 (6.68)	−.0846 (7.37)	−.0790 (6.36)	−.0793 (6.38)					
45-54	−.2290 (20.49)	−.2385 (21.40)	−.2695 (22.13)	−.2697 (22.15)					
55-64	−.4689 (43.27)	−.4789 (44.27)	−.5476 (45.55)	−.5480 (45.58)					
65+	−.9092 (87.79)	−.9208 (88.96)	−.9047 (71.80)	−.9047 (71.80)					
Black	.0355 (4.45)	.0475 (5.68)	−.0458 (5.50)	−.0459 (5.51)	−.1260 (3.07)	−.1264 (3.08)			
Asian	−.6461 (41.53)	−.7121 (42.22)	−.6574 (39.38)	−.6569 (39.34)	−.5982 (10.55n)	−.5981 (10.56)			
Native	.5352 (32.65)	.5613 (33.79)	.4468 (27.15)	.4469 (27.15)	.6269 (8.03)	.5291 (6.89)			
Hispanic	.0034 (0.36)	−.0440 (4.40)	−.0990 (9.74)	−.0991 (9.74)	−.0262 (0.76)	−.1124 (3.29)			
Other	.4615 (32.22)	.4108 (28.36)	.3525 (24.62)	.3527 (24.63)	.4719 (8.36)	.4628 (7.69)			
State	No	Yes		Yes		Yes		Yes	Yes
Education	No	No		Yes		Yes		Yes	Yes
Labor force status	No	No		Yes		Yes		Yes	Yes
Age < 25	No	No		No		No		Yes	Yes
Constant	−29.2435	−31.9401	−37.0591	1.0711	1.5404		1.6196		
Adjusted R^2^	.0734	.0807	.1035	.0445	.0779		.0802		
N	473,714	473,714	473705	473705	24377			24377	

Notes: Excluded age < 25; whites. T-statistics in parentheses. Source: BRFSS. Weighted mean = 1.24.

Li and Penn (2023) [39] found no significant difference in the raw data between whites and blacks, but we find blacks have a significantly higher number of ACEs than whites in the first column which persists as controls are added. Consistently, Slopen et al (2016) [52] found that blacks had a higher incidence of child adversity than white children. They examined racial/ethnic differences in nine ACEs (from birth to age 17 years) in the National Survey of Child Health (2011–2012) and determined how differences vary by immigration history and income (N = 84,837). This inventory included 1. financial hardship; 2. parental divorce/separation; 3. parental death; 4. parental imprisonment; 5. witness to domestic violence; 6. victim or witness of neighborhood violence; 7. lived with mentally ill/suicidal person; 8. lived with someone with alcohol/drug problem; and 9. treated unfairly because of race/ethnicity. Items 2, 4, 5, 7, and 8 were based on CDC’s BRFSS System ACE Module. Overall, 49% were exposed to at least one adversity, and 23% were exposed to two or more. Among children of U.S.-born parents, exposure to ACEs was more common among black and Hispanic children than white children: mean scores for black, Hispanic, and white children were 1.27, 1.26, and 0.90, respectively.

Giano, Wheeler and Hubach (2020) [53] examined the 2011−2014 BRFSS data used by [31] and added 11 states and updated some data but restricted the sample to a single observation per state using the latest data available for each state. They confirmed ACEs were higher for the unemployed, those unable to work as well as those with lower incomes and low educational attainment. They also found that 57.8% of individuals experienced at least one ACE. Females had significantly higher ACEs than males while White individuals had significantly lower mean ACE scores (1.53) than Black (1.66) or Hispanic (1.63) individuals. The 25-to-34 age group had a significantly higher mean ACE score than any other group (1.98). Generally, those with higher income/educational attainment had lower mean ACE scores than those with lower income/educational attainment. Sexual minority individuals had higher ACEs than straight individuals.

Overall, in our sample of 474,162 respondents, 53% of respondents said they had zero ACEs. The weighted distribution is 0 = 53%; 1 = 22%; 2 = 11%; 3 = 7%; 4 = 4%; 5 = 2%; 6 = 1%; 7 = 1%; 8 = 0.45%. In Table 5 we report four separate probit regressions with the dependent variable set to one if an individual had one or more ACEs, zero otherwise. Results are reported separately by gender for the young (age < 25) and older groups (>=25) which include a time variable set to zero in 2009 and fourteen in 2023. The probability of having one or more ACEs is rising over time for all groups, but the time coefficient is largest for young females, consistent with the significant female*time interaction term in the earlier table. Why there should have been a rise over time remains unclear especially for those over the age of twenty-five since their childhood would have ended before the start of this time-series.

**Table 5:** Determinants of adverse childhood experiences aggregate probits, BRFSS (1 if one or more ACEs, zero otherwise).

	Age < 25		Age >=25	
	Male	Female	Male	Female
Time	.0139 (2.84)	.0215 (4.12)	.0115 (9.90)	.0112 (11.24)
Black	.1664 (3.56)	.1253 (2.90)	.2026 (16.97)	.1119 (11.90)
Asian	−.4009 (7.08)	−.5271 (8.28)	−.5017 (21.41)	−.5316 (24.61)
Native	.2654 (3.22)	.1992 (2.29)	.4039 (18.11)	.2933 (14.70)
Hispanic	−.0267 (2.88)	−.0882 (2.28)	.1038 (7.611)	.0612 (4.84)
Other	.4945 (5.44)	.2202 (3.38)	.2138 (11.54)	.2123 (11.54)
Constant	−27.3577	−43.2049	−23.3796	
Pseudo R^2^	.0347	.0415	.0339	.0367
N	13,179	11198	195,814	253,514

Notes: all equations also include state, education and labor force status. 4 age dummies are in columns 3 and 4. T-statistics in parentheses.

In Table 6 we estimate probit equations for the eight ACEs for the young aged <25. Being unemployed is always significantly positive and having a college degree or more is negative and significant. For these young people the likelihood of suffering ACEs in childhood had risen over the period, apart from living with someone with a drug problem or with divorced parents. It is possible that some of this increase might have been attributable to impacts of the Great Recession or COVID, both of which may have impacted things like depression in the household. The weighted distribution is as follows – 0 = 46.5%; 1 = 22.4%; 2 = 11.7%; 3 = 7.4%; 4 = 4.7%; 5 = 3.1%; 6 = 1.5%; 7 = 1.0% and 8 = 0.6%. Weighted men = 1.24%.

**Table 6:** Age < 25 determinants of adverse childhood experiences probits.

	Depressed	Drink	Drugs	Touch sexually	Touch them	Forced sex	Hit or kicked	Divorced
Female	.3342 (19.00)	.2188 (11.91)	.1926 (9.67)	.7260 (29.23)	.6197 (23.29)	.6304 (19.90)	.2841 (14.42)	.1424 (8.32)
Time	.0329 (8.84)	.0144 (3.59)	−.0037 (0.93)	.0218 (4.27)	.0199 (3.60)	.0161 (2.45)	.0043 (1.07)	.0010 (0.28)
Black	−.4968 (14.76)	−.3052 (8.74)	−.2210 (5.98)	.1053 (2.61)	.1277 (3.01)	.1255 (2.45)	−.0093 (0.26)	.3681 (12.07)
Asian	−.6776 (13.11)	−.5387 (9.68)	−.6650 (9.56)	−.1928 (2.86)	−.2338 (2.95)	−.1539 (1.61)	.1044 (2.10)	−.4988 (10.40)
Native	.0430 (0.74)	.2876 (4.95)	.3990 (6.69)	.2131 (2.86)	.2368 (2.95)	.2595 (2.90)	.3956 (6.49)	.2279 (4.03)
Hispanic	−.3050 (11.42)	−.0726 (2.66)	−.1991 (6.57)	.1177 (3.45)	.0848 (2.30)	.1205 (2.83)	.1013 (3.53)	−.0009 (0.03)
Other	.0359 (0.86)	.1179 (2.75)	.2017 (4.648)	.2741 (5.25)	.2589 (4.66)	.3370 (5.42)	.3088 (6.95)	.2689 (6.54)
Constant	−66.7347	−19.1428	6.3924	−45.4649	−41.5418	−33.8069	−9.6242	−2.0600
Pseudo R^2^	.0438	.0317	.0404	.0866	.0761	.0768	.0308	.0507
N	24,377	24377	24,377	24,321	24,377	24,321	24,377	24,377
Mean	.32	.24	.17	.10	.08	.05	.18	.41

Notes: all equations also include education, labor force status and state dummies. T-statistics in parentheses. Excluded white

Source: BRFSS

Table 7 reports the distribution of ACEs according to whether the respondent reported being in despair. The columns present these data for three groups: young females, young males and all those aged 25 and over. Despair is much higher among those who say they had ACEs and especially so for young females. For example, 65% of young females in despair report that as a child they lived with someone who was depressed compared with 33% of those not in despair. The mean number of ACEs for young females in despair was 3.5 versus 1.7 for those not in despair. Analogously for the other two groups the probability of having a particular ACE was higher for those in despair than those who were not.

**Table 7:** Despair and ACEs by gender and age (weighted) source: BRFSS.

	Females <25		Males <25		Age>=25	
	Despair	Not despair	Despair	Not despair	Despair	Not despair
Aggregate score	3.26	1.65	2.69	1.17	2.27	1.13
Live with anyone depressed	.65	.33	.56	.23	.36	.15
Live with a problem drinker	.45	.24	.41	.19	.39	.22
Live with anyone uses illegal drugs	.39	.16	.38	.14	.23	.10
Did anyone touch you sexually	.35	.14	.16	.04	.24	.10
Anyone make you touch sexually	.27	.10	.14	.04	.20	.07
Anyone force you to have sex	.19	.07	.08	.02	.14	.04
Parents divorced/separated	.59	.42	.55	.38	.41	.27
Parent hurt you	.38	.19	.36	.21	.38	.22
N	964	10,052	704	12,261	23,913	418,307

To estimate the partial correlation between ACEs and mental and physical despair we run probit estimates in Table 8 and, below them, OLS estimates for the number of bad mental and physical health days. We do so for men and women, young and old. The aggregate ACEs score runs from zero to eight. Models contain controls for age and its square, education, race, labor force status, and state dummies. The linear time trend shows an upward trend in mental despair for all groups which is also apparent for bad mental health days. However, there are no trends in physical despair and the number of bad physical days.

**Table 8:** Mental distress and adverse child experiences, 1993-2024, BRFSS.

	Age < 25		Age >=25	
	Male (1)	Female (2)	Male (3)	Female (4)
a) Mental Despair Probit				
Aggregate score	.1937 (18.36)	.1458 (17.70)	.1322 (40.56)	.1186 (52.87)
Age	.0276 (2.54)	.0114 (1.10)	−.0063 (13.14)	−.0059 (15.42)
Time	.0190 (2.05)	.0295 (3.66)	.0058 (2.69)	−.0190 (10.95)
Pseudo R^2^	.1028	.0886	.1015	.1047
N	12,949	10,998	192,692	249,090
b) Physical Despair Probit				
Aggregate score	.0804 (5.15)	.0719 (5.37)	.0735 (22.74)	.0724 (30.92)
Age	.0283 (1.84)	.0322 (1.92)	.0106 (23.41)	.0110 (28.05)
Time	−.0033 (0.27)	.0041 (0.31)	.0007 (0.36)	−.0011 (0.70)
Pseudo R^2^	.0731	.0738	.1385	.1261
N	12,701	10,777	192,090	245,9222
c) Bad Mental Health Days OLS				
Aggregate score	1.5192 (33.58)	1.3919 (31.88)	.9268 (76.90)	.9940 (101.39)
Age	.0845 (2.12)	−.0912 (1.82)	−.0543 (37.92)	−.0667 (47.28)
Time	.2179 (7.30)	.3448 (9.82)	.0586 (9.33)	.0889 (14.80)
Adjusted R^2^	.1042	.1329	.1057	.1318
N	12,961	11,011	192,692	249,090
d) Physical Health Days				
Aggregate score	.3667 (12.80)	.3985 (14.77)	.4972 (35.38)	.5610 (53.12)
Age	.0563 (2.22)	.0385 (1.25)	.0355 (21.25)	.0385 (25.36)
Time	.0251 (1.33)	.0596 (2.75)	.0096 (1.31)	−.0098 (1.51)
Adjusted R^2^	.0327	.0419	.1475	.1541
N	12,998	11,004	192,090	247,662

Table 9 examines the correlates of mental despair in more detail. The first column, with a sample size of over six million, takes the full BRFSS sample from 2009–2024 and estimates a probit with state controls (not reported) along with year, age and its square, education, race, and labor force status. Column 3 now adds the eight ACE variables to the analysis in column 1 for all, and column 4 does so for those under twenty-five. Because these ACE variables are only available for a subset of years our sample size falls. All ACE variables are positive and statistically significant in column 3 for all. Among the young, only four of the ACEs – living with someone with depression, living with someone who drinks, being forced to have sex, and being hurt by parents – significantly adversely impacted mental health as a young adult. Despair rises in age for the young from age 18 through age 24.

**Table 9:** Mental despair and adverse child experiences, 2009-2024 probits, BRFSS.

	All	Age < 25	All	Age < 25
	2009-2023	2009-2023	2009-2023	2009-2023
ACE Depression			.3329 (39.60)	.4388 (14.51)
ACE Drink			.0918 (11.38)	.1500 (4.61)
ACE Drugs			.0984 (9.34)	.0924 (2.61)
ACE Parents divorced			.0430 (5.56)	.0275 (0.94)
ACE Touched sexually			.0948 (7.15)	.0908 (1.64)
ACE Touched them			.1010 (6.81)	.1564 (2.53)
ACE Have sex			.1634 (10.80)	.1876 (3.09)
ACE Parents hurt			.0905 (10.24)	.1311 (3.83)
Age	.0011 (33.49)		.0083 (6.52)	
Age^2^*100	−.0019 (58.77)		−.0133 (10.59)	
Age 19		.0153 (9.51)		.1544 (2.95)
Age 20		.0179 (10.96)		.2362 (4.52)
Age 21		.0200 (12.35)		.2029 (3.83)
Age 22		.0216 (13.08)		.2020 (3.69)
Age 23		.0267 (16.02)		.1689 (3.04)
Age 24		.0265 (15.70)		.2276 (4.11)
Female	.0131 (70.42)	.0282 (8.41)	.0863 (12.63)	.1825 (6.56)
Black	−.0142 (39.80)	−.0128 (8.41)	−.0508 (4.26)	−.0479 (0.96)
Asian	−.0177 (27.93)	−.0181 (9.43)	−.0970 (3.31)	−.1242 (1.48)
Native	.0122 (16.44)	−.0056 (1.88)	.0792 (3.65)	.1490 (1.77)
Hispanic	−.0101 (23.61)	−.0121 (8.57)	−.0568 (3.93)	−.0461 (1.14)
Other	.0064 (13.03)	−.0022 (1.33)	.1292 (6.94)	.0579 (0.96)
Constant	.0439	.0214	−2.1227	−2.2982
Pseudo R^2^	.0525	.0213	.1076	.1087
N	6,204,561	326,512	465,754	23,972

Contains state, year, education and labor force status dummies. Excluded whites and age 18 in columns 3 and 4.

Notes: all equations also include state, and education, race and labor force status dummies. Time = year

### 4.3. Impact of screen time and the internet

#### 4.3.1. Youth risk behavior surveillance system, 1999–2023.

In the YRBS we have information for children on the decline in their wellbeing over time. In addition we have data on internet use and on whether they were bullied and if they were forced to have sex when they were a child. All contribute significantly to feelings of sadness and hopelessness which have been on the rise since around 2010.

The YRBS first asked a question on sadness and hopelessness in 1999 that is not dissimilar to the distress and despair variable used earlier from the BRFSS.

*Q18. During the past 12 months did you ever feel so sad or hopeless almost every day for two weeks or more in a row that you stopped doing some usual activities? Yes/No*

Table 10 shows that, just as with despair, sadness and hopelessness has trended up alarmingly, especially for high school women, from 36% in 1999 to 53% in 2023, down slightly from 57% in 2021. This is consistent with data from the NHIS on sadness which is one of the six components of the Kessler score reported above. The mean score rose from 2011 as follows: 2011 = .39; 2012 = .42; 2013 = .46; 2014 = .45; 2015 = .44; 2016 = .46; 2017 = .46; 2018 = .48; 2021 = .55. It should be noted that Udupa et al (2022) pointed out that the smallest rise over time in the six components they observed through 2018 was in sadness.

**Table 10:** Sadness and screen time – Youth Risk Behavior Surveys, 1999-2023. a) % Sad or Hopeless almost every day for two weeks.

	Male	Female
1999	.21	.36
2001	.22	.35
2003	.22	.35
2005	.20	.37
2007	.21	.36
2009	.19	.34
2011	.21	.36
2013	.21	.39
2015	.20	.40
2017	.21	.41
2019	.27	.47
2021	.29	.57
2023	.28	.53

Twenge, Martin and Campbell (2018) [54], examined data from the YRBSS 8^th^, 10^th^, and 12^th^ graders 1991–2016 in the US and found that teens who spent more time on electronic communication and screens and less time on non-screen activities, had lower psychological well-being. Twenge and Martin (2020) [18] extended that work and examined smart phone use with the YRBSS for the years 2009–2015. First, they found in their Table 1 that electronic device usage was higher for males (2.07 hours) than for females (1.72 hours). They also looked at the sadness variable in YRBSS and found “*digital media time was more strongly associated with low well-being among girls than among boys, particularly for smartphone and social media use*”.

We extend that work through 2021 as screen usage pushes upwards and especially so in 2019 and 2021. There is no data on screen time use in the 2023 survey. There are three different questions asked over time, with slightly different wording covering a) 2007–2019 b) 2003–2005 and c) 2021. The first grouping covering seven surveys asks as follows.

*Q19. On an average school day, how many hours do you play video or computer games or use a computer for something that is not schoolwork? (Count time spent playing games, watching videos, texting, or using social media on your smartphone, computer, Xbox, PlayStation, iPad, or other tablet.)*

The 2003–2005 surveys ask:

*Q20. On an average school day, how many hours do you play video or computer games or use a computer for something that is not schoolwork?*

While the 2021 survey asks a broader question including watching TV:

*Q21. On an average school day, how many hours do you spend in front of a TV, computer, smart phone, or other electronic device watching shows or videos, playing games, accessing the Internet, or using social media (also called “screen time”)? (Do not count time spent doing schoolwork.)*

They all have six responses of <1 hour; 1 hour; 2 hours, 3 hours, 4 hours and 5 or more hours. There is also a code in 2021 survey which says, “*I do not play video or computer games or use a computer for something that is not schoolwork*”. The weighted distribution by year for girls and boys is reported in Table 11 with zero percent in that first category in 2021. The proportion reporting at least four hours a day rose sharply from 2011 and especially so for women. The proportion in the top two categories is especially high in 2021, presumably in part as it broadened the question to include watching TV. It is crucial in what follows to include a 2021, year dummy to control for this difference, which is always highly significant.

**Table 11:** Screen time by year (%) youth risk behavior surveys, 1999-2021.

	0 hours	< 1 hour	1 hour	2 hours	. 3 hours	. 4 hours	5 + hours
Female							
2003	28	26	14	15	8	4	4
2005	32	26	14	13	8	4	3
2007	24	24	16	15	9	5	7
2009	22	25	17	16	10	5	6
2011	15	23	18	18	11	6	10
2013	17	15	13	15	12	8	21
2015	22	11	10	14	13	9	21
2017	25	10	9	13	12	8	22
2019	23	10	9	14	14	10	21
2021	0	4	4	13	18	18	43
Male							
2003	13	23	18	20	12	6	10
2005	13	24	18	19	12	6	9
2007	13	22	17	19	13	6	10
2009	13	23	18	18	12	6	10
2011	11	18	17	19	14	8	14
2013	12	16	13	16	14	9	19
2015	14	17	11	18	14	9	18
2017	14	14	12	17	14	9	20
2019	12	11	11	18	17	11	20
2021	0	8	5	14	21	17	36

The YRBSS also contains some data on ACEs, this time whether the student was bullied or cyber bullied or was forced to have sex, with 2023 numbers in parentheses.

*Q22. During the last twelve months, have you ever been bullied on school property? Yes/No –(*males 16.6%, females 21.9%).

*Q23. During the past 12 months, have you ever been electronically bullied (count being bullied through texting, Instagram, Facebook, or other social media)? Yes/No? *– (**males 12.0%, females 20.6%).

*Q24. Have you been physically forced to have sexual intercourse when you did not want to? Yes/No (*males 4.3%, females 13.3%).

Bullying is another example of an ACE that we specifically examined in [14] for the UK using a birth cohort and was also examined as an ACE in Blanchflower and Bryson (2024b) [15] using the 2001 Eurobarometer #56.1. In addition, Blanchflower and Bryson (2024) [15] examined other ACEs including being beaten or punched by a parent in the General Social Survey, a parent dying, financial strains on the family and so on.

Bullying in childhood lowers health and wellbeing, variously measured in adults. It also lowers life expectancy. Column 1 of Table 12 reports OLS regressions for the sadness and hopelessness variable for the years 2011–2021 and for males in column 2 and females in column 3. As our measure of screen time, we simply use two dummies to identify high usage (4 hours per day) and very high usage (5 + hours per day). We also include race, gender and education and year dummies. Column 1 indicates that sadness and hopelessness rose markedly in 2019 and 2021.

**Table 12:** Sadness and hopelessness OLS regressions, 2011-2021.

	All	Male	Female	All
Screen time 4 hr/ day	.0551 (10.31)	.0432 (6.31)	.0655 (7.93)	.0380 (5.18)
Screen time 5 + hr/day	.1076 (27.39)	.0930 (17.79)	.1172 (20.01)	.0862 (15.51)
Female	.1472 (46.13)	.1343 (34.76)		
Female* screen 4 hr/day	.0356 (3.37)			
Female* screen 5 + hr/day	.0416 (5.46)			
Forced to have sex	.2323 (38.14)	.1949 (17.85)	.2456 (32.36)	.2320 (38.08)
Bullied at school	.1645 (35.40)	.1651 (25.66)	.1636 (24.54)	.1647 (35.44)
Electronic bullied	.1907 (37.54)	.1960 (25.23)	.1883 (27.52)	.1903 (37.47)
10^th^ grade	.0244 (5.54)	.0308 (5.29)	.0184 (2.81)	.0246 (5.58)
11^th^ grade	.0390 (8.87)	.0530 (9.16)	.0257 (3.88)	.0394 (8.95)
12^th^ grade	.0332 (7.45)	.0629 (10.68)	.0042 (0.64)	.0336 (7.54)
Ungraded	.0192 (0.46)	.1080 (2.14)	−.1049 (1.49)	.0178 (0.42)
Asian	−.0417 (2.56)	.0049 (0.24)	−.0971 (3.79)	−.0414 (2.54)
Black	−.0554 (3.63)	−.0342 (1.78)	−.0836 (3.47)	−.0553 (3.63)
White	−.0581 (3.90)	−.0273 (1.46)	−.0974 (4.14)	−.0578 (3.88)
Other	.0114 (0.76)	.0210 (1.12)	−.0067 (0.28)	.0114 (0.76)
2013	.0057 (1.07)	−.0016 (0.23)	.0121 (1.50)	.0049 (0.92)
2015	.0028 (0.53)	−.0162 (2.33)	.0208 (2.63)	.0018 (0.34)
2017	.0140 (2.61)	−.0079 (1.11)	.0353 (4.42)	.0133 (2.48)
2019	.0679 (11.89)	.0506 (6.69)	.0847 (9.95)	.0674 (11.80)
2021	.0783 (14.14	.0322 (4.51)	.1257 (14.83)	.0770 (13.91)
Constant	.1334	.1220	.3015	.1403
Pseudo R^2^	.1522	.0895	.1350	.1526
N	76,284	37,962	38,322	76,284

Notes: T-statistics in parentheses. Sample is YBRSS 2011–2021. Excluded Native Americans and 9^th^ grade and 2011.

Screen time is associated with greater sadness and hopelessness, and the effect rises markedly with time spent in front of a screen as indicated by the doubling of the coefficient on 5 + hours versus 4 hours a day. Women are more prone to sadness and hopelessness than men, but this is compounded by time in front of a screen, as indicated by the female interactions with screen time in column 4.

Importantly, Table 12 estimates an overall sadness and hopelessness equation for 2011–2021 that includes controls for screen time usage *and* for bullying and sexual abuse (n = 76,284). All are significantly positive. It seems that rising screen usage along with adverse childhood experiences are playing a significant part in explaining the rise of mental ill-being among young men and especially young women.

A recent IPSOS survey (n = 1607) for Pew Research found that nearly half (46%) of US teens ages 13–17 in 2022 had been bullied or harassed online, with older teens especially likely to be targeted. Offensive name calling being most commonly reported (32%) followed by false rumors (22%). In total, 28% of teens in the survey experienced multiple types of cyberbullying. (https://www.pewresearch.org/internet/2022/12/15/teens-and-cyberbullying-2022/)

As noted by CDC (2024) [55] surprisingly given the rise we have seen in screen time usage in the same data file, there has not been evidence of a *rise* in most of these three variables since 2008. The exception is the rise in being forced to have sex for females. Their estimates that we replicate are below.

**Table:** 

	Males		Females	
	2011	2021	2011	2021
Bullied	18	13	22	17
Electronically bullied	11	11	22	20
Forced to have sex	4	4	12	14

We also have a longer time run for being forced to have sex since 1999 for males with female (weighted) estimates in parentheses 1999 = .052 (.12); 2000 = .051 (.10): 2003 = .061 (.12); 2005 = .042 (.11); 2007 = .045 (.11); 2009 = .045 (.10); 2011 = .045 (.12); 2013 = .042 (.10); 2015 = .031 (.10); 2017 = .035 (.11); 2019 = .034 (.11); 2021 = .036 (.14), 2023 = 64.3(.13).

The decline of bullying is interesting as is the lack of change in the electronic bullying variables over time, but it seems that it is the usage that matters. Of note here is that in 2021 approximately 14% of high school girls said they had been forced to have intercourse against their will. This contrasts in the BRFSS with 8% of females and 3% of males ages 18–24 who said they had been forced to have sex in the period 2020–2023.

## 5. Discussion

Mental ill-health, on a variety of measures and across several data sets is on the rise in the United States and elsewhere. Of particular concern is the dramatic increase since 2011 in mental ill-being among those under the age of twenty-five and especially so for young women. By 2023 more than one in ten young women say that every day of their lives is a bad mental health day, up from 4.8% in 2010 and 3.2% in 1993 [15].

As Twenge and Farley [56] have noted there are a number of reasons why social media and Internet use might be more strongly associated with girls’ well-being than boys’ (excluding citations).

*“Girls focus more on social relationships and popularity especially during the adolescent years. Girls’ friendships tend to be both more intimate and more fragile. Perhaps as a result, the authors note, adolescent girls’ moods are more influenced by interpersonal events than boys’ are. Girls may be more likely to engage in social comparison and more likely to seek feedback on social media and spend more time carefully crafting their online image and soliciting help from friends in doing so, possibly as a result of girls self-objectifying more (placing more emphasis on how their physical bodies appear to others). Social media use is linked to worries about body weight among adolescent girls. In addition, girls may be more likely to engage in relational aggression (centered on social rejection and relationships and boys in physical aggression, a difference seemingly exacerbated by media exposure.”*

**Fig 7:**
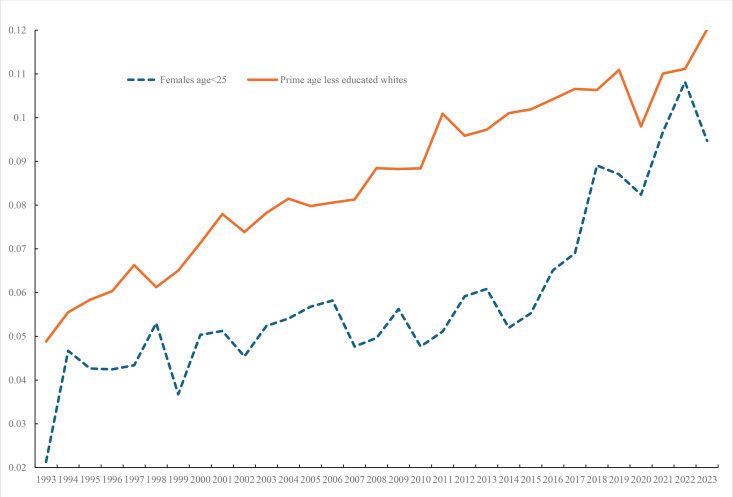
Despair for young females and prime age less educated whites, BRFSS.

The upward trend in youth ill-being is quite different from the upward trend in both the morbidity and mortality of white prime age less educated that led to a rise in deaths of despair, due to opioid overdoses, suicides and alcohol poisonings. Fig 7 compares the trends in mental despair for young women with that of white prime age non-college men. It reports despair for 1993–2023 for prime age less educated (high school diploma or less), whites ages 35–54 + 6. Also included on the graph are the data from our Fig 4 for females age < 25. Despair, measured as 30 of the last 30 days being bad metal health days, for young females has picked up sharply especially since 2011 and now despair in 2022 is approximately the same for both (10.8% for young females and 10.6% for prime age less educated whites). In past decades the advanced world had a youth unemployment problem. Now it has a burgeoning global youth mental health crisis.

Traumatic experiences in childhood appear to be having a continuing impact on the young and their deteriorating mental health. Here we find that eight ACEs relating to parental circumstances and being abused in childhood raise the probability of poor mental well-being, whether measured as bad mental health days or distress or sadness and hopelessness. Of course, youngsters under age twenty-five experienced these childhood events most recently and could still be experiencing them, for example if a parent was depressed, drinking or if they were divorced. They have an impact also on physical health but to a lesser degree.

Although we show the more ACEs reported by people, the poorer their mental health, our BRFSS data are not sufficient to estimate whether ACEs have contributed directly to the rise in poor mental health. They may have done so if the number of ACEs reported has risen over time or if sensitivity to having had one or more ACEs (captured in coefficients over time in mental health equations) has risen. Unfortunately, the BRFSS isn’t well suited to that given the way the data are collected as there is not a long run of years – we only have data for 2009–2012 and 2019–2022.

Young women are especially likely to report ACEs. The impact of being under the age of eighteen and living in a household where someone is depressed seems especially important. Of those young females who said they were in despair in the years 2020–2022 four out of five of them reported having one or more ACES: 35% had 3 or more. Of those who did not report being in despair two out of ten had one or more ACEs and 12% had 3 or more.

We also found using data on high school students ages 14–18 that there has been a marked rise in screen time which appears to be an important determinant of rising mental ill-being over and above being bullied, cyberbullied or sexually abused at school. Being bullied and abused and spending long hours on the internet all appear to lower wellbeing especially for young women.

The broad impact of these ACE variables at all ages on numerous outcomes is perhaps surprising. The issue is whether these variables fundamentally alter the life course in a bad way. Alternatively, they are simply identifying a series of characteristics that predict poor adult outcomes. The question is why and what is the mechanism? Are these ACE variables simply picking up some susceptibility in childhood to subsequent bad youth outcomes or are they having direct adverse effects? Of course, both of these things could be happening. Of course there are other possible mediating factors (e.g., social isolation, changes in parenting styles) that could be playing a role. The other issue is how accurate is the respondent’s recall? One possibility is that there is a deep endogeneity going on. People experiencing bad times may well want to blame them on their childhood experiences. Perhaps the memories are false, and this is not the case, but we are unable to disentangle such a possibility.

An obvious issue is the extent to which the relationship between smart phone use and poor mental health is causal. There is a growing recent literature summarized by Pugno (2025) [57], suggesting that indeed the relationship is causal. He cites studies for the US [58], the UK [59], Germany [60], Italy [61] and Spain [62] based on ‘natural experiments’, based on comparing a sample of the population that has access to social media to another very similar sample that does not. Braghieri et al [58] make use of evidence on the spread of Facebook across US college campuses, while the remaining studies [59–62] make use of data on the spread of broadband by area and show these have a sizeable and negative impact on youth wellbeing, and that of young women in particular. This seems to settle the matter. See also [63] McClean, Rausch and Haidt (2025) arguing these studies establish causality.

Finally, in a recent paper [1] we speculated what might lie behind the growth in poor mental health among the young in the last decade or so. One of our hypotheses was the growth in exposure to social media linked to increased screen time usage. In this paper using recent data on high school students, we have confirmed that this is one of the contributory factors since screen time, and especially lengthy screen time exposure, has been rising and is significantly correlated with poor mental health. Adverse child experiences also seem to contribute significant negative impacts later in life. The effect is particularly pronounced for young women where the growth in poor mental health has been most dramatic. This is an issue that requires urgent attention both by academics and policymakers.

## Supporting information

S1 File Appendix. Distribution of responses to Age aggregates by state, BRFSS, 2009–2024.
(DOCX)
