# Supplementary material for: The consequences of abuse, neglect and cyber-bullying on the wellbeing of the young
Source: PLoS One. 2025 Aug 19;20(8):e0327456. doi: 10.1371/journal.pone.0327456 (PMC12364343; doi:10.1371/journal.pone.0327456)
Supplement: S1 File — Distribution of responses to Age aggregates by state, BRFSS, 2009–2024. (DOCX) [file pone.0327456.s001.docx]

Appendix. Distribution of responses to Age aggregates by state, BRFSS, 2009-2024

2009 2010 2011 2012 2013 2019 2020 2021 2022 2023 2024 Total

Alabama 0 0 0 0 0 5,383 4,271 3,679 71 0 0 13,404

Arizona 0 0 0 0 0 0 7,559 2 0 0 0 7,561

Arkansas 3,460 0 0 0 0 0 0 3,863 3,843 135 0 11,301

Delaware 0 0 0 0 0 2,913 0 0 0 3,176 0 6,089

DC 0 3,332 0 0 0 0 2,374 123 0 0 0 5,829

Florida 0 0 0 0 0 11,746 7,775 43 8,623 9,156 2044 37,547

Georgia 0 0 0 0 0 0 6,224 243 0 5,685 133 12,285

Hawaii 0 5,929 13 0 0 0 6,574 0 0 0 0 12,516

Idaho 0 0 0 0 0 0 4,700 0 0 0 0 4,700

Indiana 0 0 0 0 0 6,914 16 0 0 0 0 6,930

Iowa 0 0 0 6,058 0 7,876 7,704 7,506 7,247 147 0 36,538

Kentucky 0 0 0 0 0 0 2,194 881 0 0 0 3,075

Louisiana 7,779 20 0 0 0 0 0 0 0 0 0 7,799

Michigan 0 0 0 0 0 6,945 1,868 0 0 0 0 8,813

Minnesota 0 0 12,948 0 0 0 0 0 0 0 0 12,948

Mississippi 0 0 0 0 0 4,156 5,070 3,772 307 0 0 13,305

Missouri 0 0 0 0 0 5,752 7,637 0 0 5,418 108 18,915

Montana 0 0 8,794 0 0 0 5,303 2 0 0 0 14,099

Nevada 0 3,364 0 0 0 0 1,438 1,567 2,524 1,965 165 11,023

New Hampshire 0 0 0 0 0 0 0 5,053 18 0 0 5,071

New Jersey 0 0 0 0 0 0 0 0 0 6,344 215 6,559

New Mexico 0 0 0 0 0 4,639 260 0 0 0 0 4,899

North Carolina 0 0 0 9,875 252 0 0 0 0 0 0 10,127

North Dakota 0 0 0 0 0 4,380 3,710 5,068 3,558 305 0 17,021

Oklahoma 0 0 0 3,171 336 0 0 0 0 0 0 3,507

Oregon 0 0 0 0 0 0 0 4,089 4,467 4,550 0 13,106

Pennsylvania 0 0 0 0 0 4,754 406 0 0 0 0 5,160

Rhode Island 0 0 0 0 0 4,673 4,177 12 0 4,370 202 13,434

South Carolina 0 0 0 0 0 5,018 3,288 6,609 214 0 0 15,129

South Dakota 0 0 0 0 0 0 5,574 0 6,003 344 0 11,921

Tennessee 0 0 0 5,269 0 4,033 417 0 0 4,108 0 13,827

Texas 0 0 0 0 0 0 7,457 0 0 0 0 7,457

Utah 0 0 0 0 0 0 8,550 554 0 0 0 9,104

Vermont 0 6,200 6,372 0 0 0 0 0 0 0 0 12,572

Virginia 0 0 0 0 0 7,521 7,081 5,608 8,627 4,999 0 33,836

Washington 0 0 13,138 0 0 0 0 0 0 0 0 13,138

West Virginia 0 0 0 0 0 4,493 0 0 0 0 0 4,493

Wisconsin 0 4,001 4,221 4,200 290 3,620 4,115 4,634 158 0 0 25,239

Wyoming 0 0 0 0 0 0 3,885 0 0 0 0 3,885

Total 11,239 22,846 45,486 28,573 878 94,816 119,627 53,308 45,660 50,702 1027 474,162
